# Supplementary material for: Cultivation Factors That Affect Amyloid-β Aggregation Inhibitory Activity in Perilla frutescens var. crispa
Source: Foods. 2023 Jan 20;12(3):486. doi: 10.3390/foods12030486 (PMC9913978; doi:10.3390/foods12030486)
Supplement: Supplementary file 1 [file foods-12-00486-s001.zip › foods-2165714-supplementary.pdf]

Supplemental information

Cultivation factors that affect amyloid-β aggregation inhibitory activity in *Perilla frutescens* var. *crispa*

Keiya Shimamori [1], Tomohiko Nambu [1], Daiki Kawamata [1], Masahiro Kuragano [1],
Naoki Nishishita [2], Toshifumi Iimori [1], Shinya Yamanaka [1], Koji Uwai [1] and Kiyotaka Tokuraku [1,*]

1. Graduate School of Engineering, Muroran Institute of Technology, Muroran 050-8585, Japan
2. Regenerative Medicine and Cell Therapy Laboratories, Kaneka Corporation, Kobe 650-0047, Japan

* Corresponding author: Graduate School of Engineering, Muroran Institute of Technology, 27-1 Mizumoto-cho, Muroran 050-8585, Japan;
Telephone: +81-143-46-5721, Fax: +81-143-46-5701, e-mail: tokuraku@mmm.muroran- it.ac.jp

Supplemental Figure

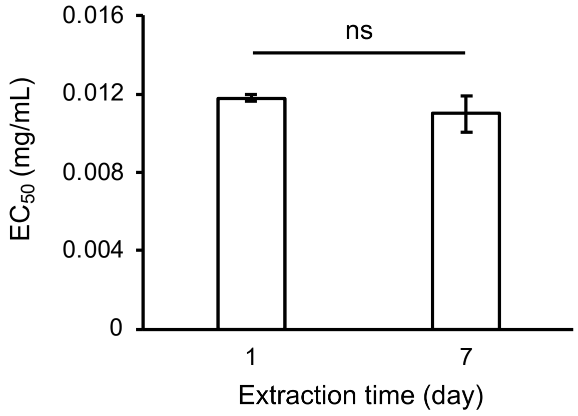

Supplemental Figure S1. Amyloid-β aggregation inhibitory activity is not changed on perilla leaf extracts within one day and seven days of extraction time. The extracts were prepared from Perilla leaves cultivated on the field in Muroran in 2020 and harvested just before flowering. The leaves were extracted for one day and seven days. EC_{50} values showing the activity were compared to extraction time. Data represent the mean ± SD (n = 3 separate plants). Comparison of EC_{50} values (ns: not significant, Welch's t-test).
